# Cardiomyogenic differentiation of an induced pluripotent stem cell line carrying PRKAG2 R302Q mutation

**DOI:** 10.1002/mco2.529

**Published:** 2024-04-19

**Authors:** Yi Gu, Xiaocheng Lu, Zuoren Yu, Jian Yao, Hongzhuan Sheng

**Affiliations:** ^1^ Department of Cardiology Affiliated Hospital of Nantong University Nantong China; ^2^ Shanghai East Hospital Tongji University School of Medicine Shanghai China; ^3^ Department of Histology and Embryology Medical School of Nantong University Nantong China

Dear Editor,

Protein kinase adenosine monophosphate (AMP)‐activated noncatalytic subunit gamma 2 (PRKAG2) cardiac syndrome (PCS), as a rare autosomal dominant genetic heart disease, is characterized by glycogen accumulation and asymmetric ventricular septal hypertrophy. Hypertrophic cardiomyopathy (HCM) usually shows left ventricle thickening, myocardial disarrangement, and an increased incidence of sudden death.^1^ It is mostly associated with genetic mutations. For example, mutations in the PRKAG2 gene, which encodes the γ2 subunit of a subunit of adenosine monophosphate‐activated protein kinase, are responsible for 0.23–1% of HCM patients.^2^ Our previous study identified the R302Q mutation in the PRKAG2 gene by sequencing analysis of 31 individuals from five generations of a family exhibiting the symptoms of ventricular septal hypertrophy.^3^ One of the family members showed the symptoms of myocardial hypertrophy and complete atrioventricular block in 1995 at the age of 25. The patient was demonstrated to have premature atrial contractions by electrocardiograph analysis (Figure 1A) and the PRKAG2 mutation (c. 905G > A, p.R302Q) by Sanger sequencing analysis (Figure 1B, upper panel). Our long‐term clinical treatment of the family demonstrated the application of β‐adrenergic receptor blockers significantly improved the prognosis of the family patients. However, its regulatory molecular mechanism remains unclear.

**FIGURE 1:**
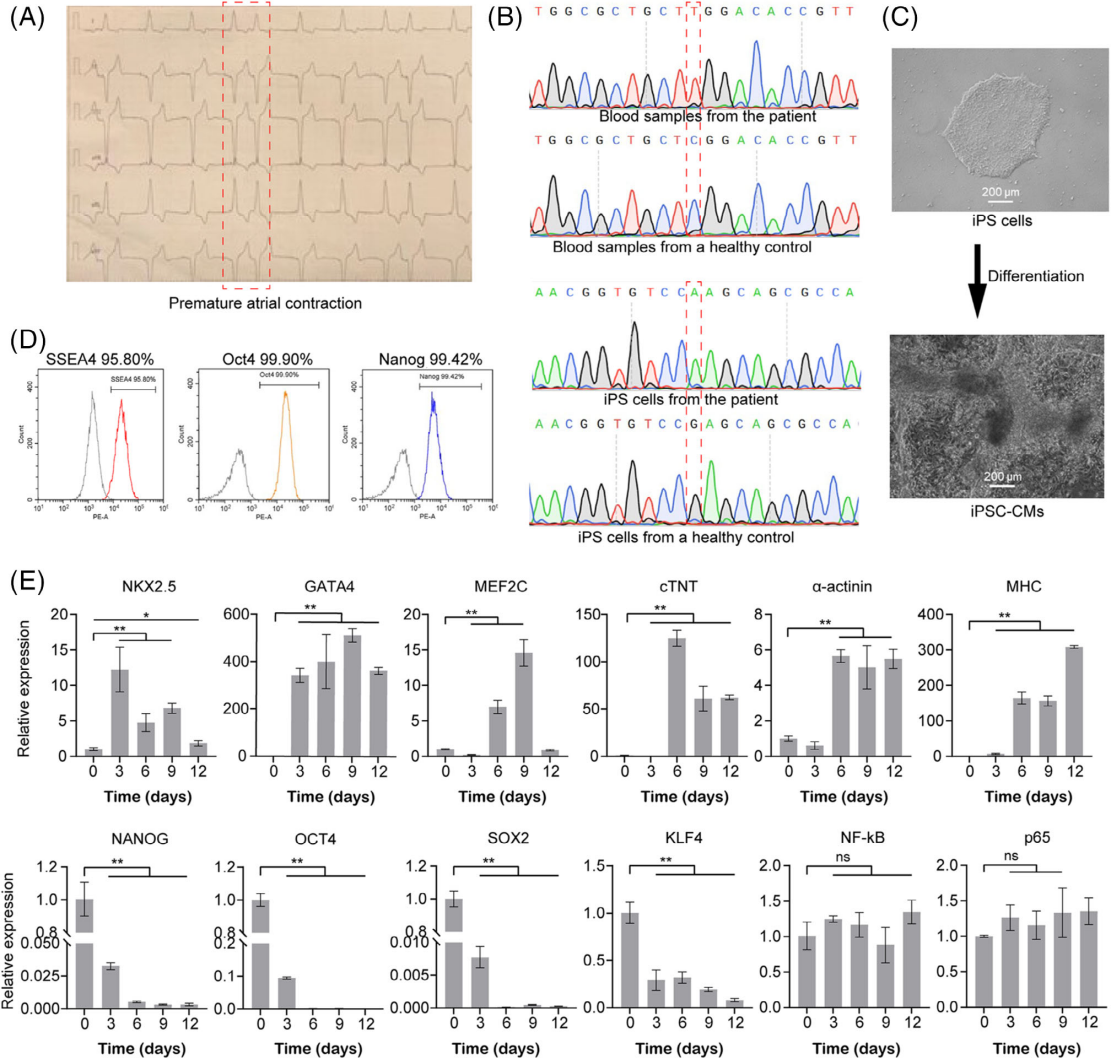
Generation of patient‐derived induced pluripotent stem cells (iPSCs), and the iPSC‐derived cardiac myocytes (iPSC‐CMs) carrying PRKAG2 R302Q mutation. (A) Electrocardiograph validation of premature atrial contractions in the patient. (B) DNA sequencing validation of the R302Q mutation (c. 905 G > A) in the PRKAG2 gene in blood mononuclear cells from the patient (upper panel) and iPSCs (lower panel). (C) Representative colony morphology image formed by the iPSCs (upper panel) and representative image of the iPSC‐CMs (lower panel). (D) Flow cytometry analysis of the iPSCs by staining stemness markers SSEA4, Oct4, and Nanog, respectively. (E) The dynamic gene expression analysis of cardiac markers (NK2 homeobox 5 [NKX2.5], GATA binding protein 4[GATA4], myocyte enhancer factor‐2C [MEF2C], cardiac troponin T [cTNT], α‐actinin, and major histocompatibility complex [MHC]), noncardiac markers (NF‐kB and its subunit p65), and stemness markers (Nanog homeobox (NANOG), Octamer‐binding transcription factor 4 (OCT4), Sex determining region Y (SRY)‐related HMG box 2 (SOX2), and Kruppel like factor 4 (KLF4)) during cardiomyogenic differentiation of the iPSCs. β‐actin was used for normalization. Data are presented as the mean ± SEM (*n* = 3). *p < 0.05, **p < 0.01.

Due to the big challenges in obtaining human cardiac cells from patients, mouse models carrying specific gene mutations have been widely used to investigate the underlying mechanisms and treatment strategies for patients with PCS and/or HCM. Transgenic mice carrying specific PRKAG2 mutations replicated similar disease phenotypes in patients. However, differences in ion channel roles, calcium handling, cardiac development, and electrophysiological properties between mice and humans indicate the necessity and importance to develop disease models using human‐origin cells.^4^ Application of the induced pluripotent stem cell (iPSC) technique has made it possible to generate patient‐derived cardiomyocytes without additional injury. These iPSC‐derived cardiomyocytes can represent myocytes in the heart of patients, holding the potential as a specific cardiac disease model in vitro for exploring molecular mechanisms and treatment strategies.^5^

Herein, we generated an iPSC cell line carrying PRKAG2 R302Q mutation using peripheral blood mononuclear cells (PBMCs) from the patient mentioned above by applying Sendai virus expressing human transcription factors Krüppel‐like factor 4 (KLF4), octamer‐binding transcription factor 3/4 (OCT‐3/4), and SRY (sex determining region Y)‐box 2 (SOX2). After 3−4 weeks of incubation, iPSCs were generated, and validated by cell colony formation assay (Figure 1C, upper panel) and flow cytometry analysis of stemness marker genes stage‐specific embryonic antigen‐4 (SSEA‐4), OCT4, and Nanog homeobox (Nanog) (Figure 1D). Chromosomal karyotyping analysis of the iPSCs revealed a normal count of 46 chromosomes and XX sex chromosomes indicating the female karyotype. No obvious abnormalities were observed in the chromosome structure. In addition, short tandem repeat assays confirmed that both PBMCs and iPSCs were from the same individual. The mycoplasma detection test demonstrated the absence of mycoplasma contamination in the iPSCs. DNA sequencing analysis of the iPSCs revealed the presence of the PRKAG2 (c. 905G > A, p. R302Q) mutation (Figure 1B, lower panel), the same as that in the patient (Figure 1B, upper panel).

In order to directly differentiate the iPSCs into human cardiomyocytes carrying PRKAG2 R302Q mutation, we used the canonical/β‐catenin Wnt pathway agonist CHIR99021 to induce the iPSCs to different and form mesoderm (day 0−3), which was validated by a significant upregulation of the mesoderm markers mix paired‐like homeobox (MIXL1) and mesoderm posterior basic helix‐loop‐helix (BHLH) transcription factor 1 (MESP1) at day 3 after differentiation. Then, the Wnt signaling inhibitor IWP2 (inhibitor of Wnt production‐2) was applied to induce cardiac progenitor cells (day 4−6). At day 11−12 of differentiation, mature cardiomyocytes were generated (Figure 1C, lower panel) with regular beating behavior. Meanwhile, cardiac markers were detected at the different stages to monitor the degree of differentiation, including the early‐stage cardiac marker NK2 homeobox 5 (NKX2.5), the middle‐stage cardiac markers GATA binding protein 4 (GATA4) and myocyte enhancer factor‐2C (MEF2C), and the late‐stage cardiac markers cardiac troponin T (cTNT), α‐actinin, and major histocompatibility complex (MHC) (Figure 1E, upper panel). As a control, the noncardiac marker gene nuclear factor kappa B (NF‐κB) and its subunit p65 did not show changes in expression during the cardiac differentiation (Figure 1E, lower panel). In addition, the expression levels of cell stemness markers NANOG, OCT4, SOX2, and KLF4 were analyzed before and after cardiac differentiation of iPSCs, indicating a significant decline along with the cardiac differentiation (Figure 1E, lower panel).

In summary, an iPSC line carrying human PRKAG2 R302Q mutation was generated using the peripheral blood mononuclear cells of a patient. It successfully differentiated into human cardiomyocytes that exhibited beating behavior and cardiomyocyte‐specific gene expression patterns. The current study not only demonstrated a valuable approach to obtain human cardiomyocytes from patients but also provided a model enabling us to better understand the underlying pathogenesis mechanisms and seek therapeutic strategies.

## AUTHOR CONTRIBUTIONS

Y.G. and X.L. performed the iPS reprogramming and cell differentiation experiments. Y.G. and Z.Y. analyzed data, organized the figures, and wrote the manuscript. J.Y. and H.S. designed the study and revised the manuscript. All authors have read the final manuscript and approved the submission.

## CONFLICT OF INTEREST STATEMENT

The authors declare no conflicts of interest.

## FUNDING INFORMATION

This work was supported by Grant 18411965900 from the Science and Technology Commission of Shanghai Municipality.

## ETHICS APPROVAL STATEMENT

The study was approved by the Ethics Committee of the Affiliated Hospital of Nantong University (2019‐K021‐01), Jiangsu Province, China. All subjects had received the written informed consent.

## Data Availability

The data included in this study are available upon request from the corresponding authors.
